# Transient breathing dynamics during extinction of dissipative solitons in mode-locked fiber lasers

**DOI:** 10.1007/s12200-024-00106-6

**Published:** 2024-01-19

**Authors:** Zichuan Yuan, Si Luo, Ke Dai, Xiankun Yao, Chenning Tao, Qiang Ling, Yusheng Zhang, Zuguang Guan, Daru Chen, Yudong Cui

**Affiliations:** 1 Hangzhou Institute of Advanced Studies, Zhejiang Normal University, Hangzhou, 311231 China; 2 State Key Laboratory of Modern Optical Instrumentation, College of Optical Science and Engineering, Zhejiang University, Hangzhou, 310027 China; 3 Wuhan National Laboratory for Optoelectronics, Huazhong University of Science and Technology, Wuhan, 430074 China; 4 Key Laboratory of Optical Information Detection and Display Technology of Zhejiang, Zhejiang Normal University, Jinhua, 321004 China; 5 School of Physics, Northwest University, Xi’an, 710127 China

**Keywords:** Breathing soliton, Fiber laser, Dispersive Fourier transform, Q-switched instability

## Abstract

**Abstract:**

The utilization of the dispersive Fourier transformation approach has enabled comprehensive observation of the birth process of dissipative solitons in fiber lasers. However, there is still a dearth of deep understanding regarding the extinction process of dissipative solitons. In this study, we have utilized a combination of experimental and numerical techniques to thoroughly examine the breathing dynamics of dissipative solitons during the extinction process in an Er-doped mode-locked fiber laser. The results demonstrate that the transient breathing dynamics have a substantial impact on the extinction stage of both steady-state and breathing-state dissipative solitons. The duration of transient breathing exhibits a high degree of sensitivity to variations in pump power. Numerical simulations are utilized to produce analogous breathing dynamics within the framework of a model that integrates equations characterizing the population inversion in a mode-locked laser. These results corroborate the role of Q-switching instability in the onset of breathing oscillations. Furthermore, these findings offer new possibilities for the advancement of various operational frameworks for ultrafast lasers.

**Graphical abstract:**

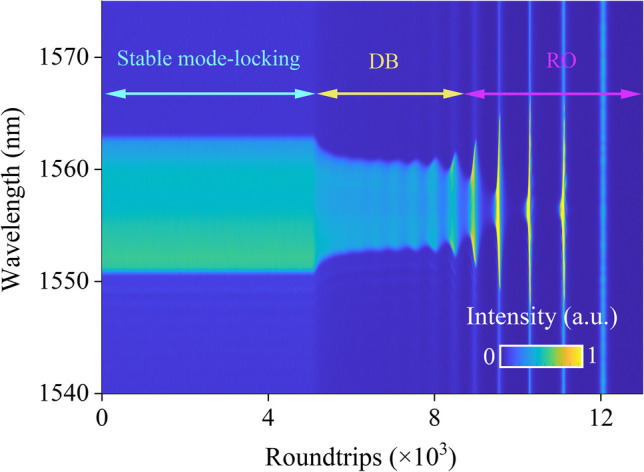

## Introduction

Passively mode-locked fiber lasers, which have intrinsic advantages such as high conversion efficiency and high beam quality, play crucial roles in various fields, including optical communications, precision spectroscopy, and industrial processing [1–5]. The ultrashort pulse generated from passively mode-locked fiber lasers is determined by the balance between various factors, including group velocity dispersion, nonlinearity, gain and loss, and birefringence [6, 7]. As a result, conventional soliton (CS) [8], dispersion management soliton (DMS) [9], dissipative soliton (DS) [10, 11], self-similar soliton [12, 13], and even birefringence-managed soliton [14, 15] can be obtained. The saturable absorber (SA), which has an ability to narrow a pulse, plays a major role in passively mode-locked fiber lasers. Commonly used SAs for mode-locking include nonlinear optical loop mirror (NOLM) [16], nonlinear polarization rotation (NPR) [17], semiconductor saturable absorption mirrors (SESAMs) [18], single-wall carbon nanotubes (SWNTs) [19–23], graphene [22, 24], black phosphorus [25], topological insulator [26] and other 2D materials [27–29]. Single-walled carbon nanotubes and graphene as SAs are used in various applications, owing to their inherent benefits of rapid recovery time and insensitivity to polarization [30, 31]. The intricate dissipative nonlinear characteristics shown by mode-locked fiber lasers enable their utilization in a broad range of application scenarios, hence enhancing the versatility of ultrafast mode-locked fiber lasers.

In recent years, with the development of emerging time-stretch dispersive Fourier transform (TS-DFT) technique, the real-time buildup dynamics of single soliton, soliton molecules and harmonic solitons have been widely observed and studied [6, 32, 33]. Mode-locking was first experimentally observed by Herink et al. [34]. This technique has the advantage of an ability to reveal various behaviors such as transient spectral broadening, wavelength shift, and beating interference pattern in a single-shot spectrum captured at a maximum frame rate of 90 MHz. Further investigation has revealed that the complete formation of an individual soliton is subject to diverse trajectories prior to reaching a stable state, owing to the influence of external disturbances [35, 36]. The independent buildup dynamics of counter-propagating pulses from modulation instability can be observed when operating a fiber laser in bidirectional mode [37]. In addition, the automated starting dynamics of the Mamyshev oscillator, which is regarded as an efficient way to generate high energy pulses, has recently been studied [38, 39].

Apart from these buildup processes of solitons, the extinction process of solitons has also received wide attention. For instance, Wang et al. experimentally observed decaying evolution processes of double-pulse mode-locking in a single-walled carbon-nanotube-based Er-doped fiber laser [40]. The results demonstrated that two pulses in one cluster disappear either simultaneously or one by one. Due to the dispersion conditions, the extinction processes of CS, DMS, and DS experience different dynamics and energy fluctuations [36]. The CS vanishes quickly, while DMS and DS experience a long Q-switched fluctuation before extinction. For the extinction processes of Mamyshev oscillator, a double Q-switching state can also be observed [39]. The decaying process from the 2nd harmonic mode-locking to the fundamental mode-locking reveals the presence of a breathing behavior [41]. Since the pump power changes significantly during the extinction process, we speculate whether breathing dynamics can occur during the annihilation of solitons. At present, there is a lack of comprehensive research on the transient breathing dynamics and the impact of breathing characteristics during the extinction process.

In this work, we have employed a comprehensive approach, integrating experimental and numerical methodologies, to investigate the transient breathing dynamics of dissipative solitons as they undergo extinction in a mode-locked fiber laser. The findings indicate that the breathing dynamics significantly influence the extinction phase of both steady-state and breathing dissipative solitons. The duration required for transient breathing dynamics demonstrates a significant level of responsiveness to changes in the pump power. Numerical simulations are employed to generate comparable breathing dynamics within the context of a model that incorporates equations describing the population inversion in a mode-locked laser. The findings presented in this study provide more evidence for the involvement of Q-switching instability in the initiation of breathing oscillations. Moreover, our findings present novel prospects for the progression of diverse operational frameworks for ultrafast lasers.

## Experimental setup

**Fig. 1:**
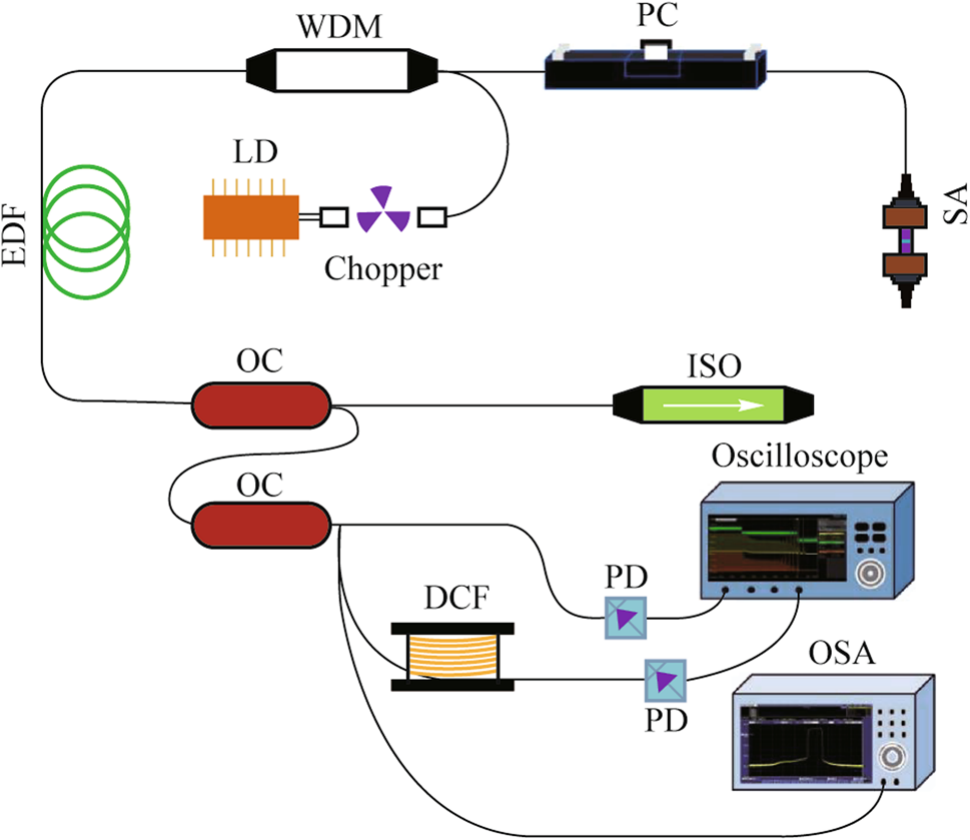
Schematic diagram of the experimental setup for passively mode-locked fiber laser and corresponding measurement system

Figure 1 illustrates the cavity structure of a passively mode-locked fiber laser as well as the corresponding DFT-based measurement system. The passively mode-locked fiber laser consists of a 980 nm laser diode (LD) with a chopper, a 980 nm/1550 nm wavelength division multiplexer (WDM), and an isolator (ISO) for unidirectional operation. The turn-off state of chopper is equivalent to decreasing the pump power to zero. The output pulses are obtained by the utilization of a 90/10 optical coupler (OC). The inside polarization controller (PC) is essential for optimizing the linear birefringence of the cavity and for manipulating the state of the laser cavity. As a gain medium, a 4.8 m erbium-doped fiber (EDF) with an absorption coefficient of 6 dB/m at 1530 nm is utilized. All the remaining fibers are classified as standard single-mode fibers (SMFs). The total length of the cavity is 7.1 m, and the fundamental repetition rate is estimated to be 28.74 MHz. Concurrently, it can be observed that the net group dispersion velocity of the laser cavity is approximately 0.046 ps^2^. This result suggests that the fiber laser operates in a normal dispersion regime. The saturable absorber (SA) uses a home-made composite film composed of polyvinyl alcohol and carbon nanotubes (PVA-CNT). The non-saturable loss, modulation depth, and saturation intensity for PVA-CNT composite film are measured as 61.2%, 12.3%, and 9.34 MW/cm^2^, respectively. An optical spectrum analyzer (OSA) and a high-speed real-time oscilloscope (20 GSa/s sampling rate) with a 5 GHz-bandwidth photodetector (PD) are used to record the time-averaged spectral and real-time temporal detections, respectively. In addition, the acquisition of real-time spectra is possible using a high-speed oscilloscope (OSC) equipped with 5 GHz PDs. A 5 km-long dispersion compensated fiber (DCF) is used to stretch the resulting pulse. The dispersion coefficient of DCF is estimated to be roughly − 160 ps/(km·nm).

## Experimental results

**Fig. 2:**
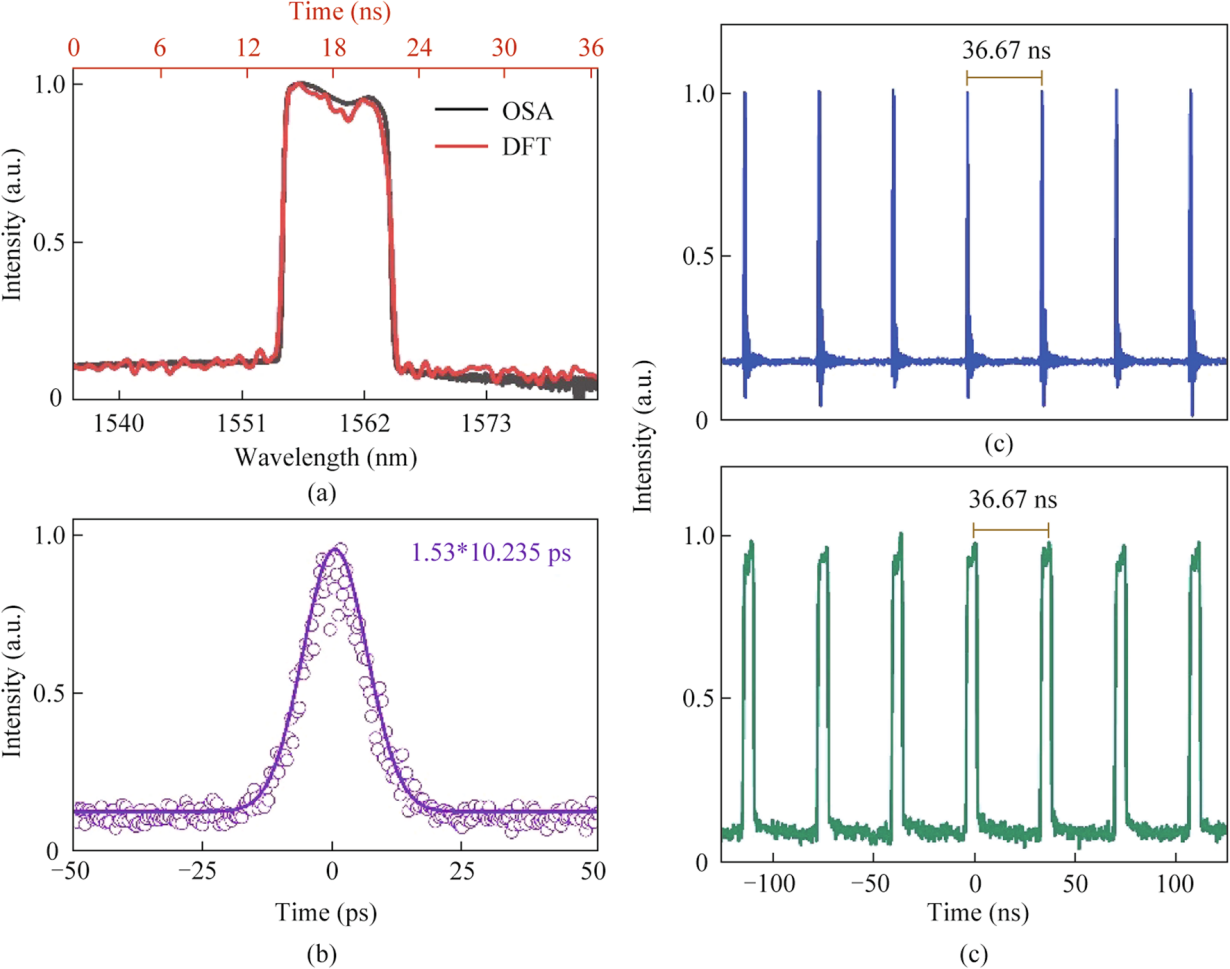
**a** Average spectrum recorded by OSA and single-shot spectrum by DFT; **b** autocorrelation trace of the pulse; **c** typical oscilloscope trace; **d** corresponding stretched pulses captured by DFT

By setting the pump power above 64 mW and optimizing the polarization state, it is possible to generate a stable dissipative soliton in the fiber laser. The average spectrum of the obtained pulse is represented by the black line in Fig. 2a, displaying a central wavelength of ~ 1557.52 nm and a 3 dB spectral bandwidth of ~ 12.54 nm. Moreover, the individual results acquired using the DFT technique are shown by the red line in Fig. 2a and demonstrate significant agreement with the results produced through traditional OSA. The measured autocorrelation trace of the pulse is shown in Fig. 2c, indicating that the pulse width is 10.235 ps. The mode-locked pulse sequence, recorded by the photodetector, is depicted in Fig. 2c. The pulse interval is approximately 36.67 ns, which is equivalent to a fundamental repetition rate of 28.74 MHz. Figure 2d depicts the time series, and shows the spectrum information obtained by the DFT. As pump power decreases from a certain threshold to zero, the stable condition of mode-locking gradually deteriorates, allowing for the observation and analysis of both temporal and spectral properties. Therefore, the data that have been obtained can contain the whole range of decay of the pulse mode-locking phenomenon, starting from a state of stability and progressing toward final annihilation.

**Fig. 3:**
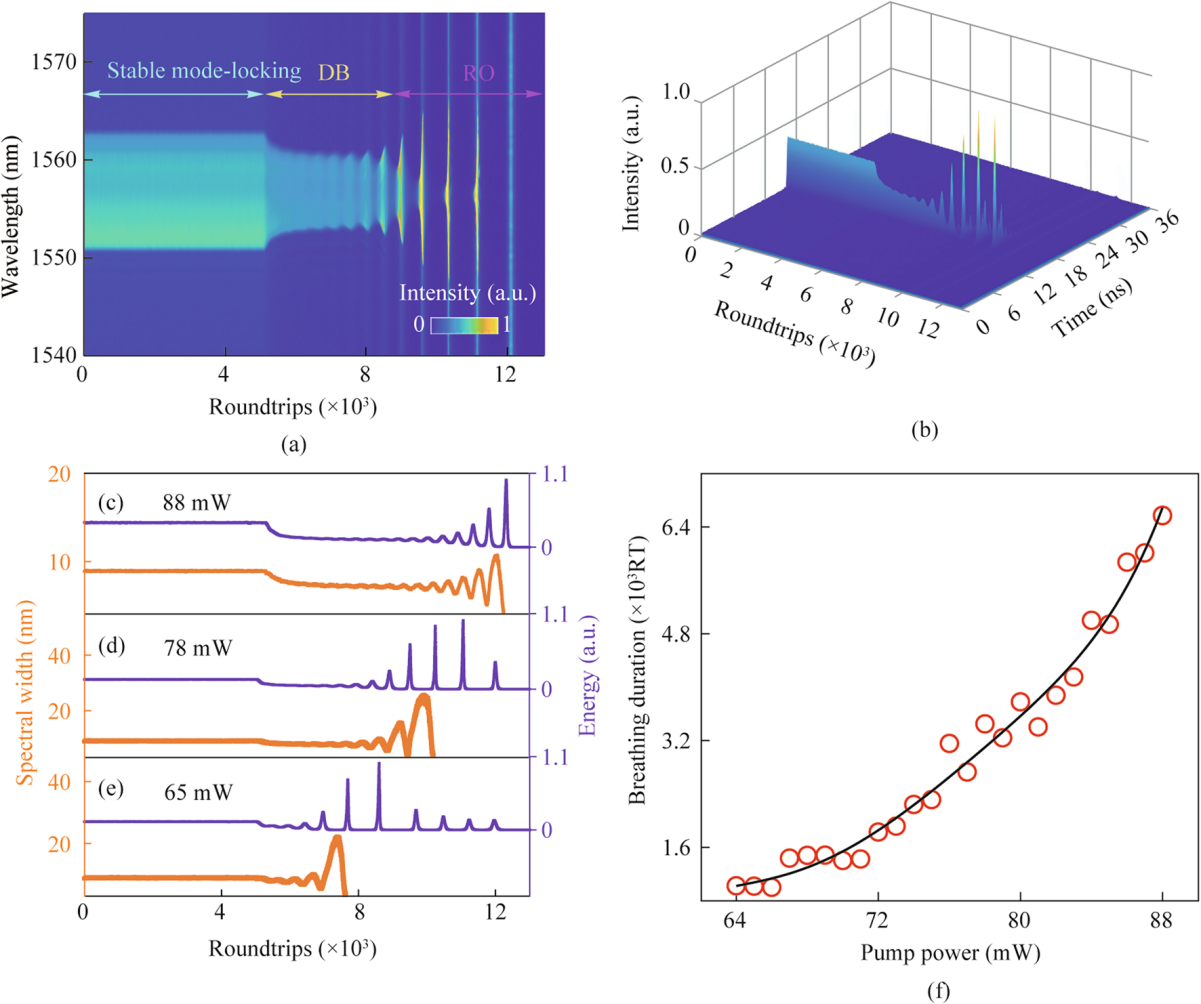
**a** Spectral decaying dynamics of stable dissipative soliton recorded by the DFT technique under the pump power of 78 mW; **b** temporal decaying dynamics of stable dissipative soliton. The corresponding calculated spectrum bandwidth and total energy evolution at pump power of: **c** 88 mW; **d** 78 mW and **e** 65 mW; **f** breathing duration as a function of pump power

To observe extinction dynamics, it is important to initially establish stable dissipative solitons. Subsequently, the chopper is closed, resulting in the pump power being reduced to zero, so facilitating the extinction of dissipative solitons. Figure 3a illustrates the entire spectral decay evolution dynamics of a steady-state dissipative soliton when the pump power is 78 mW. The result shows the three stages involved in the vanishing process, namely stable mode-locking, decaying breathing (DB), and relaxation oscillation (RO). Figure 3b displays the corresponding temporal evolution of the extinction process. In this context, it is evident that solitons exhibit a steady alteration of total energy as they dissipate. The evolution of spectral bandwidth and total energy which is extracted from the result in Fig. 3a is depicted in Fig. 3d. It can be illustrated that upon closing the chopper, the spectral width and energy undergo an initial phase of exponential attenuation, followed by a subsequent phase characterized by decaying breathing oscillation. During this latter phase, the amplitude of the breathing steadily rises. In the end, when entering the RO phase, the spectral width cannot be extracted because there is no complete pulse formation. It can be demonstrated through the analysis of total energy evolution that a perpetual oscillatory phenomenon emerges. The lack of pumping power prevents the sustained formation and dissipation of pulses, despite the presence of a continuous oscillation process.

In our experiment, dissipative solitons continue to exist even when the pump power is modified within a certain range. Nevertheless, decaying dynamics vary significantly. The entire process of decaying dynamics is similarly divided into three stages each with different pump power. However, the evolution of three stages is dissimilar. The evolutions of extracted spectral bandwidth and total energy following the disappearance of stable dissipative solitons at pump powers of 88 and 65 mW are depicted in Fig. 3c and e, respectively. From Fig. 3c, it is evident that while the alteration of pump power has little effect on the whole duration of the decaying process, it does impact the DB process more obviously. Due to the decreased inversion population, the duration for overall extinction processes is reduced to some extent. The duration time of the DB process increases as the pump power is increased, and it experiences a reduction when the pump power is decreased. This phenomenon can be linked to insufficient saturated gain, resulting in a greater buildup of population inversion. It eventually results in the further increase of the pulse energy with the emergence of self Q-switching operation. Figure 3f illustrates the changes in duration as a function of pump power during the DB phase. It may be seen that the duration time of the DB process exhibits an exponential relationship with pumping power. Figure 3f also provides a visualization of the reduction in pump power from 88 to 64 mW and the corresponding decrease in the breathing stage from 6.4 × 10^3^ roundtrips (RTs) to 1.6 × 10^3^ RTs.

**Fig. 4:**
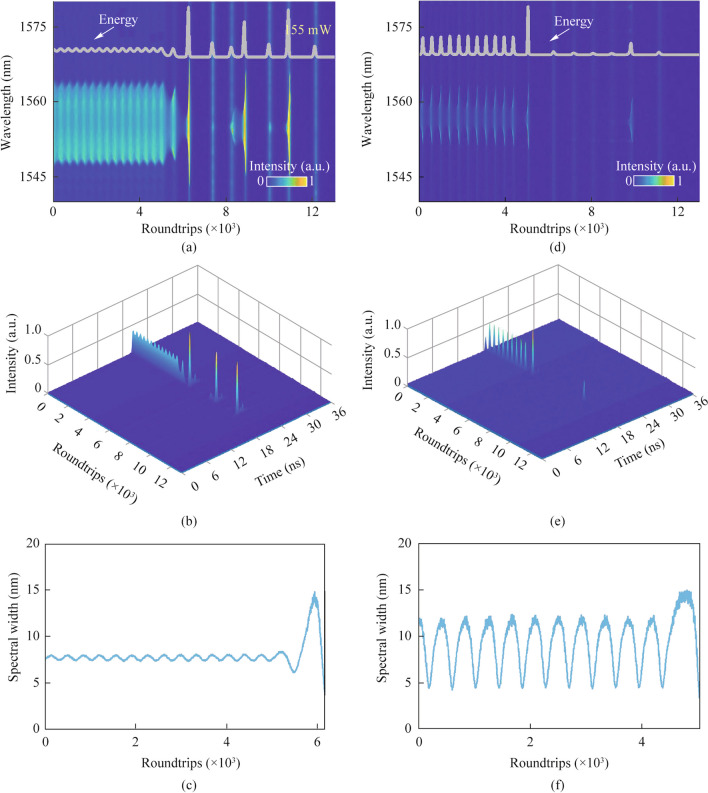
Spectral decaying dynamics of breathing dissipative soliton under the pump power of: **a** 58 mW and **d** 52 mW; temporal decaying dynamics under the pump power of **b** 58 mW and **e** 52 mW; the corresponding calculated spectrum bandwidth under the pump power of **c** 58 mW and **f** 52 mW

In a broader context, the generation of breathing solitons can be observed as a consequence of the Hopf bifurcation phenomenon when the pump power is decreased [42]. Although breathing solitons have been extensively studied [42–45], these efforts have focused on the evolution and regulation of steady-state breathing solitons. Hence, we proceed to decrease the pump power in order to investigate the annihilation dynamics of breathing-state dissipative solitons. Figure 4a and b illustrate the spectral and temporal evolution dynamics during the extinction process of breathing dissipative solitons, which were created under a pump power of 58 mW. As seen in the image, the annihilation process, akin to that for the stable dissipative soliton, may be observed to occur in three distinct stages. The distinction lies in the fact that the breathing dissipative soliton exhibits vibrational characteristics, resulting in a shortened DB phase during which the pump power remains low. Support for this statement may be derived from the evolution of spectral bandwidth, as seen in Fig. 4c. Based on the provided information, it is evident that a decrease in pump power to 52 mW results in the generation of breathing dissipative solitons exhibiting a greater breathing amplitude, as seen in Fig. 4d and e. Currently, the generation of the DB process is challenging, as seen in Fig. 4f. Nevertheless, based on the energy development graphs depicted in Fig. 4a and d, it can be seen that the RO phenomenon persists and has a prolonged duration.

## Simulation results

To verify the breathing dynamics during the decaying process, a physical model has been developed incorporating the use of rate equations, to simulate the dynamics of breathing [46, 47]. The propagation of the pulses in the EDF segments of the laser cavity is governed by the complex Ginzburg–Landau equation for a slowly varying envelope amplitude [48, 49],

1 $$\frac{\partial u}{{\partial z}} = \frac{g}{2}u - {\text{i}}\frac{{\beta_2}}{2}\frac{{{\partial^2}u}}{{\partial {t^2}}} + {\text{i}}\gamma {\left| u \right|^2}u + \frac{g}{{2\Omega_{\text{g}}^{2}}}\frac{{{\partial^2}u}}{{\partial {t^2}}},{\kern 1pt} {\kern 1pt} {\kern 1pt} {\kern 1pt} {\kern 1pt} {\kern 1pt} {\kern 1pt} {\kern 1pt} {\kern 1pt} {\kern 1pt} {\kern 1pt} {\kern 1pt} {\kern 1pt}$$

where *t* and *z* are the time and propagation distance; *β*_2_ and *γ* represent the second-order dispersion coefficient and cubic refractive nonlinearity of the fiber, respectively; *g* is the gain coefficient and Ω_g_ is the gain spectral bandwidth. Further, the power distribution along EDF is governed by the following two-level rate equations [50, 51]:

2 $$\begin{gathered} \frac{{{\text{d}}{I_{\text{s}}}(z)}}{{{\text{d}}z}} = {\Gamma_{\text{s}}}[\sigma_{\text{s}}^{\text{(e)}}{N_2} - \sigma_{\text{s}}^{\text{(a)}}{N_1}]{I_{\text{s}}}(z),{\kern 1pt} {\kern 1pt} \hfill \\ \hfill \\ \end{gathered}$$

3 $$\frac{{{\text{d}}{I_{\text{p}}}(z)}}{{{\text{d}}z}} = {\Gamma_{\text{p}}}[\sigma_{\text{p}}^{\text{(e)}}{N_2} - \sigma_{\text{p}}^{\text{(a)}}{N_1}]{I_{\text{p}}}(z),{\kern 1pt} {\kern 1pt} {\kern 1pt} {\kern 1pt}$$

4 $$\frac{{{\text{d}}{N_1}}}{{{\text{d}}t}} = {\Gamma_{21}}{N_2} + [\sigma_{\text{s}}^{\text{(e)}}{N_2} - \sigma_{\text{s}}^{\text{(a)}}{N_1}]{\phi_{\text{s}}} - [\sigma_{\text{p}}^{\text{(e)}}{N_2} - \sigma_{\text{p}}^{\text{(a)}}{N_1}]{\phi_{\text{p}}}{, }$$

5 $$\frac{{{\text{d}}{N_2}}}{{{\text{d}}t}} = - {\Gamma_{21}}{N_2} - [\sigma_{\text{s}}^{\text{(e)}}{N_2} - \sigma_{\text{s}}^{\text{(a)}}{N_1}]{\phi_{\text{s}}} + [\sigma_{\text{p}}^{\text{(e)}}{N_2} - \sigma_{\text{p}}^{\text{(a)}}{N_1}]{\phi_{\text{p}}}{.}$$

Here *I*_s_(*z*) and *I*_p_(*z*) are optical intensities of the signal and pump at position *z*, the respective photon fluxes being *φ*_s,p_ = *I*_s_/*hv*_s,p_; *σ*_p_^(a/e)^ are the absorption/emission cross sections for the pump at 980 nm; *σ*_s_^(a/e)^ are the same characteristics for the signal at 1560 nm; *N*_1_ and *N*_2_ represent the population densities of the ground and excited states; Γ_21_ ≡ 1/*τ* is the probability of the spontaneous transition from the excited state to the ground state, with respect to time *τ*; Γ_s,p_ are modal overlap factors. *N*_1_ and *N*_2_, can be calculated, as functions of *z* during one roundtrip time, from Eqs. (4) and (5), the total population being *N* = *N*_1_ + *N*_2_. Then, intensities of the signal and pump at a given position *z* can be obtained from Eqs. (2) and (3), and these values are used to calculate the population at the next position. The so obtained solution is further used to produce the one generated by the next roundtrip. The parameters of the gain medium are taken from Refs. [50–52], as given by manufacturers of the optical devices used in the setup. The parameters of the gain medium are as following: $\sigma_{\text{s}}^{\text{(e)}}$ = 5.3 × 10^−25^ m^2^; $\sigma_{\text{s}}^{\text{(a)}}$ = 5.5 × 10^−25^ m^2^; $\sigma_{\text{p}}^{\text{(a)}}$ = 3.2 × 10^−25^ m^2^; *N* = 5.4 × 10^24^ m^−3^; *τ* = 12 ms; Γ_s,p_ = 0.4.

Equations (2)–(5) are solved by means of the standard Runge–Kutta algorithm. As a result, the gain coefficient distributed along the EDF has been found, according to *g*(*z*) = (*I*_s_(*z*))^−1^d*I*_s_(*z*)/d*z* [46, 47]. The gain coefficient in Eq. (1) can then be replaced by the obtained results. Next, the action of the SA in the laser cavity is modeled by a transfer function *T* = 1 − *α*_0_/(1 + *P*/*P*_sat_) [53], where *α*_0_ is the modulation depth; *P* is the instantaneous pulse power; *P*_sat_ is the saturation power. The simulations are initiated with a weak Gaussian pulse (with peak power less than 1 × 10^−9^ W). It should be noted that no matter whether the initial condition is a weak Gaussian pulse or white low-amplitude white Gaussian noise, the result for the steady-state soliton is the same. The dispersion coefficient *β*_2_ for SMF and EDF are − 21.6 and 20 ps^2^/(nm·km), respectively. The calculated nonlinear coefficients are *γ* = 4.5 and 1.3 W^−1^·km^−1^ for EDF and SMF, respectively. The following parameters are used to produce the breathing soliton: Ω_g_ = 40 nm; *c* = 3 × 10^8^ m/s; *α*_0_ = 0.2; *P*_sat_ = 30 W.

**Fig. 5:**
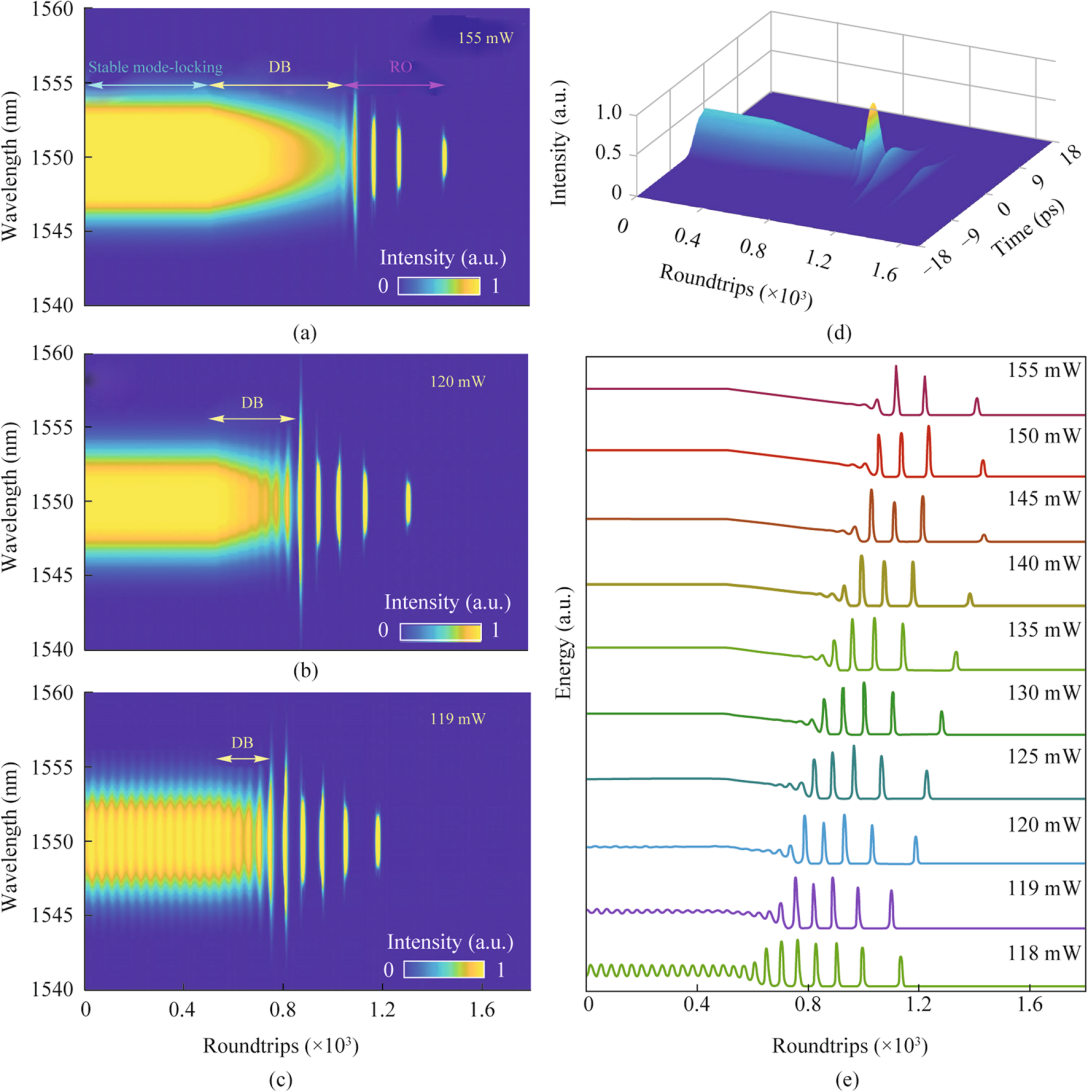
Spectral evolution of dissipative soliton with pump power of: **a** 155 mW; **b** 120 mW and **c** 119 mW; **d** corresponding temporal evolution of dissipative soliton under the pump power of 155 mW; **e** total energy evolution of dissipative soliton under different pump powers

It is important to acknowledge that there may be discrepancies between the theoretical pump power and the actual pumping power owing to variations in optical fiber specifications and practical experimentation. Stable dissipative solitons can be achieved when the pump power reaches a value of more than 155 mW. Figure 5a and d depict the theoretical simulation of the spectral and temporal extinction dynamics of stable dissipative solitons. The figures indicate a satisfactory agreement between the numerical result and the experimental findings. Furthermore, the extinction process exhibits three distinct stages that bear resemblance to one another. When the pump power is decreased to 120 mW, it is evident that the duration of the DB stage experiences a notable reduction as shown in Fig. 5b. Furthermore, the generation of breathing dissipative solitons can occur when the pump power reaches a value of 119 mW as depicted in Fig. 5c. The duration of the DB phase is 99 RTs. Figure 5e illustrates the variation of total energy throughout the process of extinction dynamics, for various pump powers. The graphic illustrates a steady reduction in the duration of the DB stage when the pump power is decreased. When the pump power decreases progressively, the breathing amplitude of the DB stage exhibits a steady rise.

Through a comparison of experimental findings and simulations, we are able to demonstrate that the rate equation accurately reproduces both breathing solitons and transient breathing dynamics. This evidence suggests a connection between breathing solitons and Q-switched instability [31]. In general, Q-switching operation and mode-locking operation can be obtained in ultrafast lasers. In addition to Q-switching and mode-locking, a SA can produce Q-switched mode-locking by emitting a bunch of mode-locked pulses with stable Q-switching envelopes. This regime could also be addressed as Q-switched instabilities, which occur when the pulse energy is temporarily increased by noise fluctuations and strong saturation of the SA. Higher saturation also reduces the tendency for Q-switching instabilities because of thermal effects or two-photon absorption, which is more significant for femtosecond pulses. Q-switching instabilities occur when the pulse energy is temporarily increased because of noise fluctuations in the laser, and these instabilities then get further increased because of the stronger saturation of the saturable absorber. This has to be balanced by a stronger saturation of the gain. If the gain is not sufficiently saturated, then the pulse energy will increase further and self Q-switching occurs.

## Conclusions

In brief, our research involves the utilization of both experimental and numerical methods to investigate the dynamics of extinction evolution in dissipative solitons within passive mode-locked fiber lasers. These solitons exhibit both steady-state and breathing-state features. The results obtained during the study suggest that the transient dynamics of breathing significantly contribute to the extinction of dissipative solitons. In addition, the duration of the transient breathing dynamics stage is highly susceptible to variations in pump power. The numerical simulations performed on a model of a mode-locked laser exhibit behaviors that align with the experimental results. This discovery has the potential to significantly advance our understanding of laser dynamics and offers novel opportunities for the development of diverse operational frameworks within the field of ultrafast laser systems.

## Declarations

### Availability of data and materials

The data that support the findings of this study are available from the corresponding author, upon reasonable request.
